# Radiation-Induced Immune Modulation and Inflammatory Responses in Human Cells and Tissues

**DOI:** 10.3390/ijms27052441

**Published:** 2026-03-06

**Authors:** Ming Chen, Nouman Amjad, Yujian Wu, Zhaojian Sun, Yirong Sun

**Affiliations:** 1 Provincial Key Laboratory of Stem Cell and Regenerative Medicine, Guangdong Hong Kong Joint Laboratory for Stem Cell and Regenerative Medicine, Guangzhou Institutes of Biomedicine and Health, Chinese Academy of Sciences, Guangzhou 510530, China; chen_ming@mail.ustc.edu.cn (M.C.); nouman@gibh.ac.cn (N.A.); wu_yujian@gibh.ac.cn (Y.W.); 2 Division of Life Sciences and Medicine, University of Science and Technology of China, Hefei 230026, China; 3 Department of Pathology, Faculty of Veterinary and Animal Sciences (FV&AS), The Islamia University of Bahawalpur, Bahawalpur 63100, Pakistan; 4 GMU-GIBH Joint School of Life Sciences, Guangzhou Medical University, Guangzhou 511436, China; sun_zhaojian@gibh.ac.cn

**Keywords:** radiation, immune modulation, inflammatory response, radiotherapy, radiation injury

## Abstract

Radiation exposure from environmental sources, medical procedures, or space exploration poses considerable risks to human health, with profound effects on immune function and inflammatory responses. Radiotherapy (RT) is a cornerstone of modern cancer treatment, leveraging ionizing radiation to induce DNA damage and tumor cell death. However, its biological effects extend beyond direct cytotoxicity, exerting complex and context-dependent influences on both innate and adaptive immunity. Ionizing radiation can enhance antitumor immune responses by promoting tumor antigen release, activating dendritic cells, and augmenting cytotoxic T-cell priming. Conversely, it can also induce immunosuppressive mechanisms, including lymphocyte depletion, regulatory T-cell expansion, immune checkpoint upregulation, and chronic inflammatory signaling, which may limit therapeutic efficacy. These immune effects are critical for optimizing RT protocols, particularly in the era of immunotherapy, where immune modulation plays a pivotal role in treatment efficacy. This review summarizes the current knowledge concerning how radiation induces immune and inflammatory responses in cells and tissues; focuses on key molecular pathways such as the DNA damage response, cGAS–STING signaling, and immune checkpoint modulation; and discusses their clinical implications. These findings provide potential therapeutic strategies for cancer treatment by harnessing the immunomodulatory potential of radiation while reducing adverse effects and for the prevention and treatment of radiation-related diseases.

## 1. Introduction

Radiation exposure, whether from natural sources, RT, or accidental nuclear events, remains a marked concern for human health [1]. Among its myriad effects, ionizing radiation can impair the vitality and function of highly sensitive hematopoietic, intestinal, and immune systems, severely disrupt immune homeostasis and weaken the body’s ability to mount an effective immune response [2,3,4]. Understanding these effects is vital for medical applications, radiation protection, and disaster response. This review explores how radiation influences immune-related gene expression, cellular responses, tissue damage, and long-term health implications [5,6,7].

Exposure to radiation, such as X-rays and gamma rays, can have complex and often destructive effects on human cells and tissues through direct ionization and the indirect generation of reactive oxygen species (ROS) [6,8]. It leads to damage to DNA and cellular structures, triggering the apoptosis or necrosis of immune cells. Lymphocytes, including T cells and B cells, are particularly vulnerable because of their high turnover rate and active DNA repair mechanisms [9,10]. Acute exposure to radiation can rapidly deplete these cells, weaken adaptive immunity and increase susceptibility to infections. Moreover, radiation can disrupt cytokine signaling and communication between immune cells, further impairing immune surveillance against pathogens and malignant cells [11,12].

Radiation can trigger a series of immune and inflammatory responses that vary with radiation dose, duration, and tissue type [12,13,14]. Bone marrow, the spleen, and the thymus, which are central to immune cell production and maturation, become significantly damaged. Bone marrow, which is responsible for hematopoiesis, experiences reduced blood cell production, leading to leukopenia and thrombocytopenia. Chronic exposure exacerbates these effects, potentially causing immune deficiency disorders or autoimmune responses due to altered self-tolerance mechanisms [15,16,17].

The immune system’s response to radiation also involves inflammation and oxidative stress, which can lead to secondary tissue damage [18,19,20]. While low doses may induce adaptive responses, high doses overwhelm repair mechanisms, resulting in irreversible harm. Medical applications, such as radiotherapy for cancer, exploit these effects to target tumors, but unintended immune suppression can complicate treatment outcomes [21].

Understanding these effects is crucial for mitigating risks in contexts such as space travel, nuclear accidents, and RT [22,23]. By studying these immune effects, we aim to develop strategies for treating radiation damage and improving RT methods [24,25]. This knowledge is indispensable for safeguarding public health in an era where radiation exposure is becoming increasingly prevalent [26,27]. In this review, we integrate evidence from radiation experimental models, clinical studies, and transcriptomic analyses to provide a comprehensive perspective on how radiation exposure affects the regulation of immune signaling pathways, immune cell dynamics, tissue damage, and long-term health outcomes.

## 2. Radiation Dose and Biological Effects

The biological effects of radiation involve primarily cellular damage through direct and indirect mechanisms. In the direct mechanism, radiation acts directly on key biomolecules such as DNA, resulting in strand breaks, base damage, or cross-linking; in the indirect mechanism, radiation ionizes water molecules to produce free radicals, which then attack DNA, proteins, or lipids, causing oxidative damage. The complex relationship between radiation dose and biological immune effects has become a key area of research, particularly in the context of cancer treatment and radiation protection. This relationship is characterized by a dose-dependent response, which can either enhance or suppress immune function, with profound implications for treatment outcomes and patient prognosis [6,28].

### 2.1. Low-Dose Radiation-Induced Immune Modulation and Adaptive Responses

Low-dose radiation (LDR) exposure has been shown to induce distinct biological effects that differ markedly from high-dose responses [2,29]. These effects include the bystander effect, in which unirradiated cells respond to signals from irradiated neighbors, and hormesis, which is a beneficial adaptive response at low doses [30]. LDR can also trigger adaptive responses, enhance cellular repair mechanisms and potentially offer protective benefits against subsequent high-dose exposure. These phenomena challenge the traditional linear no-threshold (LNT) model, suggesting that low doses may not always be harmful and could even stimulate beneficial immune responses under certain conditions [29,30]. Studies have shown that human monocytes exposed to 0.05 and 0.1 Gy irradiation (IR) exhibit notable upregulation of Toll-like receptor (TLR) signaling molecules, including high-mobility group box 1 (HMGB1), TLR4, TLR9, MyD88, and IRAK1, suggesting the activation of innate immune signaling pathways at low radiation doses [31].

Studies using mouse models of pancreatic cancer have demonstrated that localized low-dose IR (0.5–2 Gy) can reprogram the tumor microenvironment (TME). This reprogramming promotes the polarization of tumor-associated macrophages (TAMs) toward an inducible nitric oxide synthase (iNOS)-expressing M1 phenotype, enhances the expression of vascular markers such as CD31 and VCAM-1, and stimulates the production of chemokines that facilitate the recruitment and infiltration of effector T cells into the tumor [32]. These results have also been validated in pancreatic adenocarcinoma patients receiving neoadjuvant therapy, where a single 2 Gy dose was sufficient to increase the number of T cells in the TME [32]. For instance, studies have indicated that LDR can augment T-cell-dependent immune responses, leading to long-term immune enhancement and increased resistance to lethal high-dose IR in experimental models [31]. While LDR may trigger adaptive responses and enhance cellular repair, the LNT model’s assumption of linear risk without a threshold remains contentious. Epidemiological studies such as the International Nuclear Workers Study (INWORKS) support LNT’s cautious approach [33], but biological evidence of hormesis challenges its universality. This conflict highlights the need for more nuanced models that account for both the potential benefits and the risks of low-dose radiation.

### 2.2. High-Dose Radiation-Induced Immune Response and Tissue Injury

In contrast, high-dose radiation, such as that used in cancer RT, exerts a dual effect on the immune system [34,35]. On the one hand, it can enhance tumor immunogenicity by inducing immunogenic cell death (ICD), characterized by the release of damage-associated molecular patterns (DAMPs), promoting T-cell infiltration, and upregulating major histocompatibility complex (MHC) class I molecules on tumor cells, thereby improving antigen presentation and T-cell-mediated cytotoxicity. RT-induced antitumor immunity depends mainly on the cGAS-STING cytosolic DNA sensing pathway, which drives dendritic cells to produce radiation-induced immune signaling type I interferons, thereby activating CD8^+^ T-cell mediated specific immune responses [36]. Such effects are crucial for converting immunologically “silent” tumors into immunologically active lesions that may respond better to immunotherapy [34,37]. On the other hand, high-dose radiation can also induce immune suppression, including lymphocyte depletion and systemic immunosuppression, which may counteract its beneficial effects [21]. The balance between these conflicting immune outcomes depends on multiple factors, including the total radiation dose, fractionation scheme, and specific characteristics of the TME. The dose and fractionation scheme determine the extent of DNA damage, cell death, and immune cell depletion. Moreover, components of the TME, such as the composition of tumor-associated lymphocytes, stromal elements, and the cytokine milieu, finely tune the degree of immune activation or suppression, ultimately determining the net immune response and therapeutic outcome [38].

## 3. Pro-Immunogenic Responses to Radiation

RT stands as a cornerstone in cancer treatment, leveraging ionizing radiation (IR) to induce DNA damage in tumor cells, thereby triggering cell death or senescence. Beyond its direct cytotoxic effects, RT profoundly influences the tumor immune microenvironment, orchestrating complex signaling pathways that can either enhance or suppress antitumor immunity. Understanding these immune signaling pathways is pivotal for optimizing RT efficacy and fostering synergistic combinations with immunotherapy.

### 3.1. Radiation-Induced DNA Damage and Trigger for Immune Activation

**Figure 1:**
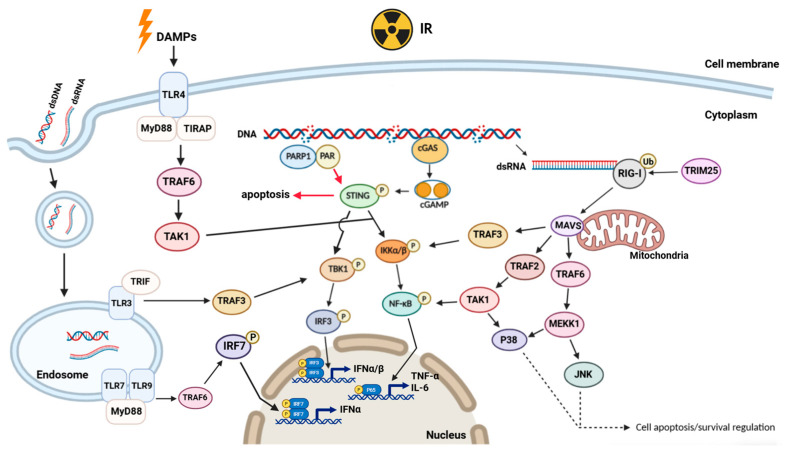
Radiation induces the activity of the innate immune signaling pathway (Created in BioRender. Long, Q. (2026) https://biorender.com/mzlrunh (accessed on 20 January 2026)).

The effects of radiation on damage to nucleic acids can be direct or indirect through free radicals [39,40]. Immune DNA damage responses (DDR) involve mainly DAMP molecules released by damaged cells, such as HMGB1, as well as RNA and DNA in the cytoplasm [39,41]. Sensors such as Toll-like receptors, RIG-1 (RNA), and cGAS–cGAMP–STING (DNA) link the DDR with pro-immune and proinflammatory responses, mainly through the TBK1/IRF3 and NF-κB pathways, activating positive and negative feedback loops in immunity, aging, autophagy, and cell death (Figure 1) [7,42].

#### 3.1.1. DAMPs

Radiation-damaged cells release DAMPs to activate innate immune cells such as neutrophils, macrophages, and dendritic cells, and can also activate nonimmune cells such as epithelial cells, endothelial cells, and fibroblasts, promoting the release of inflammatory mediators [39,42,43,44]. Endogenous DAMPs include HMGB1, extracellular cold-inducible RNA-binding protein (CIRP/CIRBP), histones, cell surface-exposed calreticulin (CALR), heat shock proteins (HSPs), ATP, nuclear DNA and mitochondrial DNA (mtDNA), extracellular RNA (exRNA), and uric acid (Table 1) [41,42].

The release of DAMPs can be divided into two main pathways: passive release and active release [41]. Passive release is associated mainly with cell death processes, including apoptosis, necrosis/programmed necrosis, pyroptosis, and ferroptosis. Moreover, DAMPs can also be actively released through secretion mechanisms, such as exocytosis in the form of lysosomes and exosomes (Figure 1) [39].

#### 3.1.2. Radiation-Induced Immune Signaling Pathways

IR triggers DNA double-strand breaks (DSBs) of nuclear DNA or mitochondrial DNA damage, which are major sources of cytoplasmic DNA and activate STING-mediated pathways [3,45,46]. When RT generates cytosolic DNA fragments, cyclic GMP-AMP synthase (cGAS) detects these fragments and synthesizes cyclic dinucleotides, which activate stimulator of interferon genes (STING) (Figure 1). STING subsequently triggers the production of type I interferon (IFN) and other inflammatory cytokines, which are pivotal for dendritic cell (DC) activation and T-cell priming [45,46]. The data also demonstrated that radiation-induced DSBs accumulation in tumor cells and their exosomes leads to the recruitment of DCs to the TME, facilitating antigen presentation and cytotoxic T-cell activation.

RT-induced DSBs activate the DDR pathway. This response involves the key protein ATM (ataxia-telangiectasia mutated), which phosphorylates downstream effector molecules such as NEMO (NF-κB essential modulator), promoting NF-κB activation in immune cells [47,48]. NF-κB is a critical transcription factor that drives the expression of proinflammatory cytokines and chemokines, thereby enhancing the maturation of antigen-presenting cells (APCs) and the activation of T cells. For example, studies using phospho-flow cytometry have shown that RT-induced activation of ATM in monocytes leads to NF-κB phosphorylation, which is crucial for APC functional maturation and the subsequent regulation of immune responses.

These pathways underscore the ability of radiation to convert “cold” tumors into “hot” tumors, characterized by increased immune cell infiltration and responsiveness to immunotherapy.

TLRs are key components of the innate immune system. In the context of radiation exposure, the activation mechanisms of TLRs and their interactions with IR have become a research focus, as radiation can induce cellular damage, release DAMPs, and subsequently trigger TLR-mediated immune responses (Figure 1) [49]. Excessive activation of TLR4 is closely associated with radiation-induced acute lung injury, through a mechanism involving the regulation of the NF-κB/NLRP3 inflammasome pathway, which is significantly upregulated in lung inflammation following radiation [50]. The effects of radiation on TLR signaling pathways are not limited to inflammatory responses but also involve the complexity of immune regulation. For example, TLR2 may mediate tissue damage following radiation exposure through the NF-κB/NLRP3 pathway, and TLR2 deficiency can alleviate inflammation, suggesting that TLRs may play a dual role in tissue repair after radiation [51]. In addition, the application of TLR agonists in immunotherapy indicates that TLRs can indirectly influence immune reconstruction after radiation by enhancing vaccine adjuvant effects or modulating the activity of lymphocytes in the tumor microenvironment [51,52]. The TLR7 (single-stranded RNA sensing), TLR3 (double-stranded RNA sensing), and TLR9 (DNA sensing) pathways activate innate immunity and potentiate anticancer responses through endosomal RNA/DNA recognition and coordinated immune signaling [53,54]. These findings reveal the multifaceted role of TLRs in radiation biology: On the one hand, TLR activation may exacerbate radiation-induced inflammation and organ damage; on the other hand, TLR-mediated immune enhancement can be used to improve immune defense or cancer treatment after radiation.

The C-terminal domain (CTD) of RIG-I acts as a sensor for RNA 5′-triphosphate (5′-pppRNA) and may be activated during radiation-induced RNA damage, causing RIG-I to transition from an autoinhibited monomeric state to a dimer, thereby interacting with the adaptor protein IPS-1 (MAVS) via the CARD domain, activating downstream NF-κB and IRF3 pathways, and inducing the expression of interferon and proinflammatory cytokines (Figure 1) [55,56]. Studies have confirmed that in A549 (NSCLC) cell lines and lung cancer tissues, the RT-induced cellular immune response relies mainly on the RNA virus sensor pathway, particularly the RIG-I–MAVS axis, rather than the previously emphasized DNA sensor cGAS-STING pathway [56]. It has also been reported for the first time that transposable elements (TEs), particularly long terminal repeats (LTRs, such as LTR21B), are key endogenous ligands that activate RIG-I after RT [56]. The expression of cold-inducible RNA-binding proteins (CIRP and RBM3) is significantly upregulated after exposure to IR, and these proteins regulate DNA damage repair, apoptosis, and radiosensitivity by activating the TLR4/NF-κB/MAPK inflammatory pathway, becoming key stress mediators and potential therapeutic targets in the radiation response [57,58].

Davide Cinat and colleagues compared the effects of photon (γ-ray) and proton radiation on mouse salivary gland organoids [59]. Both types of radiation initially cause mitochondrial DNA (mtDNA) to be released into the cytoplasm and stimulate the production of ZBP1, a cytosolic nucleic acid sensor involved in mtDNA recognition. However, proton radiation led to a more pronounced loss of heterochromatin regulatory factors at later stages postirradiation. This study also confirmed that transposable element expression was more active [56,59]. Genetic and pharmacological studies have confirmed the critical role of IFN-I signaling in enhancing the activity of salivary gland stem and progenitor cells both in vitro and in vivo [59].

Radiation affects a series of proinflammatory cytokines (such as IL-6, TNF-α, and IL-1β) and anti-inflammatory cytokines (such as IL-10), as well as chemokines (such as CCL2, CXCL8, and CXCL10), through the above signaling pathways [60]. These signaling molecules play a central role in mediating tissue inflammation, immune responses, and subsequent injury and repair by activating related signaling pathways and recruiting immune cells. The pattern of their release and the intensity of their effects are closely related to the radiation dose, type, and duration of exposure (Figure 1, Table 1).

**Table 1:** Molecular products of radiation damage.

Type of Damage	Release Molecules	Identified Receptor	Inductive Positioning	Key Cytokines Induced
DNA double-strand break	Nuclear DNA fragments, mitochondrial DNA	cGAS [61], PARP1 [4]	Cytoplasm, nucleus	IFN-I (IFN-α/β), pro-inflammatory cytokines (TNF-α, IL-6, IL-1β)
RNA cleavage/abnormal processing	5′-ppp dsRNA, ssRNA	RIG-I [55], TLR3 [54], TLR7 [53]	Cytoplasm, Endosome	IFN-I, IFN-III (IFN-λ), pro-inflammatory cytokines (TNF-α, IL-6)
Cell Stress/Death	CIRP, HMGB1, DNA-RNA hybrids	TLR4 [50], TLR9 [31]	Cell membrane, Endosome	TNF-α, IL-1β, IL-6, IL-10, IFN-γ

### 3.2. Immunosuppressive Effects of Radiation

Despite its immune-enhancing potential, radiation can induce profound immunosuppression [21]. This dual nature—RT can both enhance the immunogenicity of tumors and suppress immune responses—presents notable challenges in cancer treatment. Understanding these immunosuppressive effects is crucial for optimizing treatment strategies and mitigating adverse consequences.

#### 3.2.1. Lymphocyte Depletion and Immune Suppression

High-dose or fractionated RT regimens often lead to lymphopenia, particularly affecting CD4^+^ and CD8^+^ T cells, which play key roles in adaptive immunity. This lymphopenia is attributed to radiation-induced apoptosis of lymphoid tissue cells and disruption of immune cell trafficking pathways. Clinical studies have shown that severe lymphopenia during RT is associated with poor responses to immunotherapy, highlighting the need to optimize dosing to balance tumor control with immune preservation [21,40].

In hepatocellular carcinoma (HCC), the DNA repair protein RECQL4 inhibits the cGAS-STING pathway through RT-induced DNA damage, thereby weakening immune activation and reducing RT sensitivity [61]. This highlights RECQL4 as a potential therapeutic target to improve the synergy between RT and immunotherapy. RT also upregulates immunosuppressive factors like TGF-β and regulatory T cells (Tregs), which are often linked to the senescence-associated secretory phenotype (SASP) of irradiated tumor cells, promoting an immunosuppressive TME that counteracts the immune-enhancing effects of RT [37]. Additionally, RT affects signaling pathways such as PLC-PIP2 and cAMP-PKA. Elevated cAMP levels suppress IFN-γ secretion and enhance Treg activity, impairing cytotoxic T-cell responses [62]. Targeting pathways such as the cAMP-PKA pathway could help alleviate immunosuppression and improve immune effector function.

#### 3.2.2. Myeloid-Derived Suppressor Cells (MDSCs) and Tregs

Radiation expands MDSCs and Tregs in the tumor microenvironment, which together suppress cytotoxic T-cell function. MDSCs are recruited through exosomes and cytokines, and inhibit immunity via arginase-1, ROS, and nitric oxide. They also secrete IL-10 and TGF-β to promote Treg expansion [21,63,64]. Tregs accumulate post-radiation and further suppress effector T cells by releasing the same anti-inflammatory cytokines. These mechanisms are especially active in hypoxic tumor areas and contribute to radiation-driven immune evasion. Studies have reported that when cocultured with T cells, DCs irradiated with 0.05 Gy have the greatest proliferative capacity and increase the production of IL-2, IL-12, and IFN-γ in the supernatant of the coculture system [65].

Tregs, characterized by Foxp3 expression, maintain immune tolerance by suppressing effector T cells via cytokine production (IL-10 and TGF-β), cell–cell contact (CTLA-4 and LAG-3), and metabolic disruption (tryptophan depletion via IDO). High-dose radiation (HDR, ≥1.0 Gy) can significantly induce the production of Tregs, especially CD4^+^ CD25^+^ Nrp1 Tregs [66]. In cancer, Tregs and MDSCs engage in bidirectional crosstalk: MDSCs induce Treg differentiation, while Tregs increase MDSC survival and function, creating a synergistic immunosuppressive microenvironment that facilitates tumor progression [67].

Therapeutic strategies targeting these cells include MDSC depletion, differentiation therapy, inhibition of suppressive pathways (e.g., arginase-1, and iNOS), and Treg targeting (e.g., anti-CTLA-4 antibodies). Future efforts should focus on precision targeting, biomarker development, and elucidating signaling pathways (e.g., STAT3) to enhance immunotherapy efficacy [68]. Together, MDSCs and Tregs represent pivotal targets for restoring immune surveillance in cancer and inflammatory diseases.

Combining RT with immune checkpoint inhibitors (ICIs) may enhance antitumor immunity by leveraging RT-induced immune activation. However, overcoming immunosuppression and tumor resistance requires innovative strategies.

### 3.3. Radiation-Induced Immune Cell Dynamics

Different cells exhibit significant differences in sensitivity to radiation, depending mainly on their proliferative activity, degree of differentiation, and DNA repair capacity. This phenomenon follows the classic Bergonié-Tribondeau law [66]: cells that are actively dividing and undifferentiated are more sensitive to IR, whereas terminally differentiated, quiescent cells are more resistant to radiation. Although lymphocytes are terminally differentiated cells, they are highly sensitive “nonproliferating cells” due to the high activity of their DDR pathways. In addition, the effects of radiation are influenced by the characteristics of biological systems, including cell cycle sensitivity and radiation defense mechanisms—cells are most sensitive to radiation during the M and G2 phases but relatively resistant during the S phase [69]. Organisms mitigate radiation damage through DNA repair mechanisms (such as nucleotide excision repair) and antioxidant defense systems.

#### 3.3.1. The Dual Effects of Radiation on Immune Cells

Radiation exposure disrupts the balance of immune cell populations, particularly affecting T and B lymphocytes [21,70]. RT activates dendritic cells by releasing tumor-associated antigens (TAAs) to initiate CD8^+^ T cells, whereas it also promotes B-cell differentiation into antibody-secreting plasma cells, enhancing opsonization and complement-mediated lysis. Helper T cells secrete cytokines such as IL-2 and IFN-γ, bridging and amplifying cytotoxic T-cell activity and B-cell maturation, forming a synergistic antitumor immune response [2,71]. RT induces immunogenic death of tumor cells, releasing damage-associated molecular patterns that serve as “find me” signals to recruit lymphocytes; at the same time, RT upregulates MHC-I expression on tumor cells, enhancing antigen presentation, expanding tumor-specific CD8^+^ T-cell clones, and inhibiting regulatory T-cell activity [45,72]. However, long-term or high-dose RT can lead to T-cell exhaustion (through upregulation of PD-1 and TIM-3) and cause lymphocyte apoptosis, resulting in reduced numbers of T and B cells, with transient immunosuppression that may increase infection risk. In solid tumors, B-cell infiltration is often inconsistent [21,73].

Radiation upregulates MHC-I molecules on tumor cells, improving their recognition by cytotoxic T cells. Additionally, it enhances the generation of TAAs, expanding the antigenic repertoire available for immune surveillance [74].

DDR and cell cycle regulation exhibit subgroup-specific mechanistic differentiation in immune cell populations, profoundly affecting their functional outcomes. In CD8^+^ T cells, the ATM/ATR-p53-p21 signaling pathway triggers reversible G1/S phase arrest, shifting metabolic reprogramming toward glycolysis, and thereby enhancing proliferative capacity and cytotoxic function upon recovery [20,27,75]. In regulatory T cells (Tregs), Ku70 enhances nonhomologous end joining (NHEJ) to maintain proliferation, stabilize FOXP3, and amplify suppressive function [76]. Conversely, CD4^+^ T cells are particularly sensitive to PARP1 inhibition, leading to replication stress and G2/M phase arrest, resulting in functional exhaustion [75]. Inflammatory macrophages activate the ATM/ATR–ATF4–PDIA3 pathway without inducing cell cycle arrest, driving a proinflammatory, oxidative metabolic state and the secretion of CCL2 and ACSL4 [77]. These adaptive changes form the basis for functional specialization—Tregs leverage repair to achieve suppression, CD8^+^ T cells use arrest to enhance vigor, CD4^+^ T cells lose function when repair is impaired, and macrophages repurpose the DDR for inflammatory polarization.

#### 3.3.2. Myeloid Lineage Cells

Myeloid cells, including macrophages and neutrophils, play pivotal roles in inflammation and tissue repair. Research on rodent models has demonstrated that radiation exposure triggers significant changes in these cells within the urinary bladder, promoting inflammatory responses and tissue damage [78]. In particular, macrophages exhibit altered behavior postexposure, contributing to endothelial dysfunction and atherosclerosis-like conditions by fostering immune cell adhesion and plaque formation in arteries. These findings underscore the systemic impact of radiation on myeloid lineage cells, linking them to chronic inflammatory diseases [79].

In noncancerous tissues, radiation activates mast cells, leading to the release of inflammatory mediators such as histamine and proteases. This activation can cause barrier dysfunction in epithelial tissues, as exemplified by studies on the urinary bladder, where radiation-induced mast cell degranulation disrupts urothelial integrity. Such tissue damage can exacerbate inflammation and immune dysregulation, posing challenges in managing radiation-induced side effects [80].

## 4. Inflammatory Responses and Tissue Damage

### 4.1. Radiation-Induced Inflammatory Responses and Tissue Specific Effects

Radiation exposure triggers distinct inflammatory pathways and tissue-dependent effects, largely driven by endothelial dysfunction and immune activation. Endothelial cells lining blood vessels are highly sensitive to radiation, leading to barrier dysfunction, compromised vascular integrity, and increased adhesion of immune cells such as macrophages—initiating processes such as atherosclerosis, with even low-dose space radiation inducing similar long-term cardiovascular risks [81,82]. The inflammatory cascade is further amplified by DAMPs released from dying cells, which activate innate immune receptors (e.g., Toll-like receptors) and stimulate the production of proinflammatory cytokines such as IL-1 and TNF-α, resulting in acute inflammation characterized by vasodilation and immune cell infiltration in exposed tissues [83].

IR at doses ≥2 Gy triggers ICD in tumor cells, leading to the release of DAMPs. These DAMPs activate the cGAS-STING pathway, which subsequently induces IFN1 signaling within 2 h post-irradiation. This activation drives the upregulation of proinflammatory cytokines, including IL-1 and TNF-α, thereby linking DDRs to innate immune activation [84]. At higher doses (≥15 Gy), IR additionally induces the synthesis of large quantities of poly(ADP-ribose) (PAR) by PARP1. This PAR accumulation triggers a STING-mediated cell death pathway, contributing to intestinal tissue damage [3].

Notably, tissue-specific variations in the immune response are observed across different organ systems. In the hematopoietic system, immune cells are rapidly depleted, while excessive DAMP signaling can exhaust hematopoiesis and disrupt hematopoietic stem cell function [85]. The gastrointestinal tract experiences mucosal barrier breakdown, bacterial translocation, and injury, which are correlated with circulating mtDNA in conditions such as intestinal ischemia–reperfusion [86,87,88]. Conversely, the skin and bone marrow exhibit heightened immune reactivity, whereas the lungs and skin are prone to chronic inflammation with fibrosis [89,90]. In contrast, the central nervous system is more resistant to radiation-induced immune responses [91]. Together, these mechanisms contribute to the development of acute radiation injury and long-term risks of cardiovascular and fibrotic disorders.

RT induces immediate DNA damage in tumor cells, leading to the release of DAMPs and proinflammatory cytokines. This activates innate immune cells, including macrophages and DCs, which adopt an M1-like proinflammatory phenotype and enhance antigen presentation [21,37,45]. Studies have shown that RT upregulates MHC-I and PD-L1 expression on tumor cells, potentially priming the TME for immune checkpoint inhibitor (ICI) therapy [92]. However, this phase is often short-lived, as radiation also promotes immunosuppressive pathways [93].

### 4.2. Chronic Inflammation and Fibrosis

Chronic radiation exposure can trigger persistent inflammation and responses that ultimately drive fibrotic tissue remodeling, particularly in organs such as the bladder and lungs [93,94]. In radiation-induced cystitis, prolonged exposure leads to irreversible bladder remodeling driven by fibrosis, which is mediated by inflammatory cells such as mast cells releasing proinflammatory cytokines, amplifying tissue damage and impairing organ function. Similarly, in other tissues, radiation-induced inflammation can progress to fibrosis, characterized by excessive collagen deposition and loss of elasticity, severely disrupting physiological processes [13,95]. Long-term effects involve persistent inflammation and immune dysregulation due to epigenetic changes and SASP in irradiated cells. These changes establish a self-perpetuating inflammatory feedback loop that exacerbates tissue injury and promotes fibrotic progression over time [96].

## 5. Therapeutic Strategies and Clinical Implications

### 5.1. Protective Strategies for Radiation Injury and Therapeutic Approaches

As mentioned above, IR primarily induces DNA damage through direct ionization or the formation of free radicals, which can give rise to chromosomal aberrations and mutations. Cells typically respond by activating the DNA repair pathways [97]. Stem cells play a key role in tissue regeneration, and are highly sensitive to radiation. However, these cells are more likely to undergo apoptosis rather than repair, potentially exacerbating tissue damage [98,99]. Radiation can also trigger inflammation [80,81,82]. Understanding these mechanisms is essential for the development of effective radioprotective agents.

Current research focuses on antioxidants such as amifostine, which can mitigate side effects by scavenging free radicals and enhancing DNA repair [100,101,102] (Table S1). Such agents may help reduce inflammation and protect immune function. Additionally, anti-inflammatory drugs (e.g., nonsteroidal anti-inflammatory drugs) and cytokine modulators (e.g., G-CSF for hematopoietic recovery) can help alleviate radiation-induced damage [103,104]. Since the 1980s, advances in genetic engineering have enabled the production of recombinant cytokines such as G-CSF, GM-CSF, and TPO, which are recommended by the International Atomic Energy Agency for managing acute radiation syndrome. Among these, TPO receptor agonists like romiplostim have shown promise as radiation mitigators. Romiplostim—approved for idiopathic thrombocytopenic purpura—has been demonstrated in animal studies to improve survival and accelerate platelet recovery after lethal IR through multiple mechanisms, including the promotion of DNA repair, anti-apoptosis, ROS removal, and antioxidant pathway regulation [105]. Antiapoptotic drugs also have the potential to protect against radiation-related intestinal injury [4,88]. Moreover, emerging approaches such as stem cell therapy and immune checkpoint inhibitors aim to restore tissue function and balance immune responses, although these strategies are still under investigation [88,98,99,106,107].

Current research aims to address practical challenges such as optimal dosing, timing, long-term safety, and the need for rapid radiation-exposure biomarkers, whereas regulatory pathways such as the FDA’s “Animal Rule” facilitate the development of countermeasures where human efficacy trials are not feasible.

### 5.2. Challenges in Radiation Dose Optimization

A central challenge in modern RT is the optimization of the radiation dose to enhance pro-immunogenic effects while limiting radiation-induced immunosuppression [38,108]. This is particularly crucial in hypoxic tumor regions, which are radiation-resistant and often exhibit heightened immune suppression, necessitating strategies like enhancing oxygen delivery or the use of hypoxia-targeting agents [21,109] (Table S1). In addition, the timing and sequence of RT in combination with immunotherapy require careful consideration to prevent immune exhaustion or the induction of immune tolerance [110].

Radiation dose rate and fractionation play critical roles in shaping immune responses, with recent research underscoring that radiation dose is a key determinant of immunological effects in the tumor microenvironment [37,111]. Accordingly, tailored RT protocols are needed to optimize immune modulation while minimizing adverse effects. The integration of RT with immune checkpoint inhibitors shows promise but varies on the basis of dose and delivery parameters [112,113]. Future research should focus on elucidating in vivo dose-rate effects, developing predictive models, and incorporating immune monitoring into clinical trials to refine protocols [114]. Overall, the relationship between radiation dose and immune responses is complex and dose dependent, with low-dose exposure potentially conferring protective or immunostimulatory effects, whereas high-dose radiation exerts both immunogenic and immunosuppressive effects [109,110].

In addition, nuclear medicine and radiopharmaceuticals play key roles in promoting global health through precise diagnosis and treatment. However, infrastructure and affordability are major barriers in low-resource areas. Research on and the production of radiopharmaceuticals must prioritize green synthesis methods and cost-effective manufacturing, as well as rapid metabolism and reduced tissue damage, to meet global medical needs [115].

## 6. Conclusions

RT induces complex immunological effects through key signaling pathways, most notably the DDR, cGAS–STING activation, and immune checkpoint modulation. Depending on the dose, timing, and tumor context, these pathways can either stimulate or suppress antitumor immunity, providing a mechanistic foundation for the rational integration of RT with immunotherapeutic strategies. Increasing evidence supports the concept that radiation functions as an in situ vaccine capable of enhancing adaptive immune responses and improving the efficacy of adoptive cell-based therapies, including chimeric antigen receptor (CAR)–T and CAR–NK cell approaches. In parallel, emerging strategies involving engineered biological vectors offer additional potential by alleviating tumor hypoxia and promoting immune cell recruitment within the tumor microenvironment.

Despite these advances, optimizing radiation dosing and delivery remains a critical challenge, as the therapeutic benefit depends on achieving a balance between immune activation and radiation-associated toxicity. The identification and validation of predictive biomarkers will be essential for stratifying patients and enabling personalized radioimmunotherapy regimens.

Future research should prioritize the elucidation of additional immune-regulatory pathways, including the roles of innate lymphoid cells and key molecular targets such as RECQL4 cAMP–PKA signaling and cGAS-STING signaling, across diverse tumor types. The integration of computational modeling with multiomics profiling holds significant promise for refining radiation protocols and improving patient-specific treatment planning. Moreover, continued efforts are needed to mitigate radiation-induced immune suppression and tissue injury, including lymphocyte depletion, chronic inflammation, and fibrosis, through the development of effective radioprotective and immunomodulatory agents.

Ultimately, advancing precision-based combination strategies that synergize RT with immune modulation will be essential for unlocking the full therapeutic potential of radiation as a cornerstone of cancer immunotherapy.

## Data Availability

No new data were created or analyzed in this study. Data sharing is not applicable to this article.

## Supplementary Materials

The following supporting information can be downloaded at: https://www.mdpi.com/article/10.3390/ijms27052441/s1.

## Abbreviations

The following abbreviations are used in this manuscript:

RT	Radiotherapy
LDR	Low-dose radiation
LNT	Linear no-threshold
TLR	Toll-like receptor
ROS	Reactive oxygen species
HMGB1	High-mobility group box 1
TME	Tumor microenvironment
TAMs	Tumor-associated macrophages
iNOS	Inducible nitric oxide synthase
ICD	Immunogenic cell death
IR	Irradiation
DAMPs	Damage-associated molecular patterns
MHC	Major histocompatibility complex
IR	Ionizing radiation
DDR	DNA damage response
CIRBP	Cold-inducible RNA-binding protein
CALR	Cell surface-exposed calreticulin
HSPs	Heat shock proteins
mtDNA	Mitochondrial DNA
exRNA	Extracellular RNA
DSBs	DNA Double-strand breaks
cGAS	Cyclic GMP-AMP synthase
STING	Stimulator of interferon genes
IFN	Type I interferon
DC	Dendritic cell
APCs	Antigen-presenting cells
CTD	C-terminal domain
HCC	Hepatocellular carcinoma
Tregs	Regulatory T cells
SASP	Senescence-associated secretory phenotype
MDSCs	Myeloid-derived suppressor cells
ICIs	Immune checkpoint inhibitors
TAAs	Tumor-associated antigens
PAR	Poly ADP-ribose
CAR	Chimeric antigen receptor
