# Supplementary material for: Radiation-Induced Immune Modulation and Inflammatory Responses in Human Cells and Tissues
Source: Int J Mol Sci. 2026 Mar 6;27(5):2441. doi: 10.3390/ijms27052441 (PMC12986220; doi:10.3390/ijms27052441)
Supplement: Supplementary file 1 [file ijms-27-02441-s001.zip › ijms-4151201-supplementary.pdf]

Supplementary Table S1 Radiation Injury Treatment and Radiotherapy Optimization Methods

Category	Specific Methods/Strategies	Primary Functions/Objectives	Notes/Examples
Radiation Injury Protection & Treatment	1. Antioxidants	Scavenge free radicals, enhance DNA repair, and mitigate side effects.	e.g., Amifostine (WR-2721), an FDA-approved radioprotector for specific clinical use [100–102].
	2. Anti-inflammatory Drugs	Reduce radiation-induced inflammatory responses.	e.g., Nonsteroidal anti-inflammatory drugs (NSAIDs) [115].
	3. Cytokine Modulation	Promote hematopoietic recovery and manage acute radiation syndrome (ARS).	e.g., Granulocyte colony-stimulating factor (G-CSF), Granulocyte-macrophage colony-stimulating factor (GM-CSF), Thrombopoietin (TPO). Recommended by the International Atomic Energy Agency for ARS management [103,104].
	4. Anti-apoptotic Drugs	Protect cells from apoptosis and reduce tissue damage (e.g., intestinal injury).	Related drugs show potential.
	5. Thrombopoietin Receptor Agonists	Act as radiation mitigators, improving survival and accelerating platelet recovery.	e.g., Romiplostim, approved for idiopathic thrombocytopenic purpura. Animal studies indicate it works through multiple mechanisms including promoting DNA repair, anti-apoptosis, reactive oxygen species removal, and regulating antioxidant pathways. Other oral agents (e.g., eltrombopag, avatrombopag) have also been developed [104].
	6. Stem Cell Therapy	Aim to restore tissue function.	Still under investigation.
Radiotherapy Optimization & Combination Strategies	1. Dose & Fractionation Optimization	Enhance pro-immunogenic effects while limiting radiation-induced immunosuppression.	Radiation dose is a key determinant of immunological effects in the tumor microenvironment. Tailored radiotherapy protocols are required.
	2. Overcoming Tumor Hypoxia	Address radioresistance and immune suppression in hypoxic regions.	Strategies include enhancing oxygen delivery or using hypoxia-targeting agents.
	3. Combination with Immune Checkpoint Inhibitors	Balance immune responses to improve efficacy.	Shows promise, but outcomes vary based on dose and delivery parameters. Careful timing and sequencing are needed to prevent immune exhaustion or the induction of immune tolerance.
	4. Distinguishing Low-Dose vs. High-Dose Effects	Select doses based on therapeutic goals.	Low-dose exposure may have protective or immunostimulatory effects, while

			high-dose radiation exerts both immunogenic and immunosuppressive influences.
Nuclear Medicine & Radiopharmaceuticals	1. Green Synthesis & Cost-Effective Production	Promote global health by making diagnosis and treatment more accessible.	In resource-limited settings, infrastructure and affordability are major barriers. Prioritizing green synthesis methods and cost-effective manufacturing processes is essential.
	2. Rapid Metabolism & Reduced Tissue Damage	Improve treatment safety.	Drug development should focus on properties like rapid metabolism and reduced tissue damage.
